# Effect of different material protocols on the control of dentin hypersensitivity: a split-mouth randomized controlled clinical trial

**DOI:** 10.1007/s00784-026-06776-0

**Published:** 2026-02-28

**Authors:** Júlia Marques Martins, Maria Fernanda Ferreira Nogueira, Guilherme José Pimentel Lopes de Oliveira, Alexandre Coelho Machado, Paulo César de Freitas Santos Filho, Hugo Lemes Carlo, Carlos José Soares, Gisele Rodrigues da Silva

**Affiliations:** 1 Department of Operative Dentistry and Dental Materials, School of Dentistry, Federal University of Uberlândia, Uberlândia, MG Brazil; 2 Department of Operative Dentistry and Dental Materials, School of Dentistry, Federal University of Uberlândia, Uberlândia, MG Brazil; 3 Department of Periodontology and Implantology, School of Dentistry, Federal University of Uberlândia, Uberlândia, MG Brazil; 4 Department of Operative Dentistry and Dental Materials, Technical School of Oral Health, Federal University of Uberlândia, Uberlândia, MG Brazil; 5 Federal University of Uberlândia School of Dentistry, Avenida Pará, 1720, Bloco 4L, Anexo A, Sala 4LA33, Campus Umuarama, Uberlândia , MG 38405-320 Brazil

**Keywords:** Dental sensitivity, Quality of life, Dentin adhesives, Pain assessment

## Abstract

**Objectives:**

To compare the efficacy of three desensitizing protocols for dentin hypersensitivity (DH) pain control and their impact on oral health–related quality of life (OHRQoL) up to 180 days.

**Materials and methods:**

In this randomized controlled split-mouth clinical trial, 33 participants (99 teeth) were randomized and treated; 31 participants (93 teeth) were analyzed (mITT). Participants received three protocols: **GLU**: 3% potassium nitrate + glutaraldehyde/HEMA desensitizer (control); **GAS**: 3% potassium nitrate + glutaraldehyde/HEMA desensitizer + universal adhesive; and **GAR**: 3% potassium nitrate + glutaraldehyde/HEMA desensitizer + universal adhesive + bulk-fill flowable resin. Pain intensity was recorded using the Numeric Rating Scale (NRS; 0–10) after standardized air-blast and tactile stimuli at baseline, immediately after intervention, and at 7, 30, 90, and 180 days. OHRQoL was assessed using OHIP-14 at baseline, 90, and 180 days.

**Results:**

NRS scores decreased over time for both stimuli (air-blast: *p* < 0.001; tactile: *p* < 0.001). No differences were observed among protocols (air-blast: *p* = 0.910; tactile: *p* = 0.681) and no time × protocol interaction was detected (air-blast: *p* = 0.341; tactile: *p* = 0.738). OHIP-14 scores improved at 90 and 180 days versus baseline (*p* < 0.001).

**Conclusions:**

The three protocols produced comparable patient-centered outcomes, indicating that adding adhesive and resin steps to potassium nitrate + glutaraldehyde/HEMA does not enhance DH pain control or OHRQoL up to 180 days.

**Clinical relevance:**

Increasing complexity with adhesive and resin steps was not associated with superior patient-centered outcomes within 180 days.

**Clinical trial registration number:**

RBR-109h3wcv.

## Introduction

Dentin hypersensitivity (DH) is defined as a short and sharp pain arising from exposed dentin in response to thermal, evaporative, tactile, osmotic, or chemical stimuli that cannot be attributed to any other dental defect or pathology [1]. Although its pathophysiological mechanism is still under debate, Brännström’s hydrodynamic theory remains the most widely accepted explanation [2]. According to this theory, external stimuli cause fluid movement within exposed dentinal tubules, resulting in the activation of nerve endings located near the dentin-pulp junction, which leads to the perception of pain [3]. Therefore, two conditions must be present for DH to manifest: dentin exposure, usually due to enamel loss and/or gingival recession with concomitant cementum loss and root surface exposure [4], and permeable dentinal tubules, allowing communication between the oral cavity and the dental pulp [5].

Several etiological and predisposing factors contribute to the development of DH, with dentin exposure being the essential requirement for pain manifestation. Such exposure may result from a presumably multifactorial pathology consisting mainly of cervical abfractive, abrasive, and/or erosive components (or a combination) [6]. In these cases, the tubules are open to the oral environment and susceptible to external stimuli [7]. Among the associated etiological factors are chronic traumatic toothbrushing, occlusal overload resulting in tooth flexure, parafunctional habits, inflammatory periodontal diseases, acute trauma, periodontal surgeries, tooth malposition within the arch, and highly acidic diets [8].

The prevalence of DH varies across the population, with conservative estimates reporting rates of approximately 11.5%, while the overall average from studies is around 33.5% [9]. The pain associated with DH can impair essential oral functions such as eating and oral hygiene, negatively impacting quality of life [10–12]. Its intensity ranges from mild discomfort to persistent pain and significant emotional distress [13]. As symptoms worsen, routine activities like toothbrushing are often neglected, favoring dental biofilm accumulation and increasing the risk of caries, gingivitis, and periodontal disease [14].

The clinical control of DH is essentially based on two approaches: modulation of the neural response, aiming to reduce the excitability of nerve fibers, and occlusion of the exposed dentinal tubules. Among the agents with neural action, potassium salts, particularly potassium nitrate, are the most commonly used. These compounds act by depolarizing the cell membrane, reducing the response to painful stimuli [3]. Regarding tubule-occluding agents, Gluma desensitizer (Kulzer, Hanau, Germany), composed of 5% glutaraldehyde and 35% hydroxyethyl methacrylate (HEMA), is noteworthy. Its mechanism involves protein coagulation of the dentinal fluid followed by monomer polymerization, sealing the tubules [15, 16]. This mechanism directly contributes to reducing the fluid displacement associated with pain.

Among tubule-occluding approaches, several therapeutic strategies have been proposed to reduce dentin hypersensitivity by limiting dentinal fluid movement, including topical fluorides/varnishes and resin-based sealing materials [17]. Within this category, adhesive systems and resin-based materials can be applied to seal exposed dentinal tubules and reduce the transmission of hydrodynamic stimuli to the pulp–dentin complex. When polymerized, these adhesive/resin materials form a protective layer over the dentin surface, acting as an artificial smear that can occlude exposed tubules [18]. Furthermore, organic or polymeric materials, with or without inorganic fillers, may exhibit lower solubility in acidic environments than purely inorganic compounds, which could favor their clinical persistence [19].

Despite the wide range of available products for DH control, achieving consistent and sustained symptom relief supported by stable tubule sealing under oral challenges remains difficult [20]. Studies suggest that combining agents with distinct mechanisms of action may enhance clinical effects compared with a single active ingredient [21, 22]. In this study, the interventions were conceived as a sequential, layered surface-sealing approach applied in a single session: potassium nitrate for neural modulation, a glutaraldehyde/HEMA agent for reduction of dentin permeability, followed by thin polymeric sealing films produced by active rubbing of an adhesive and, when applicable, a small amount of bulk-fill flowable resin. Conceptually, because the outermost polymeric film is directly exposed to the oral environment, it may be more susceptible to mechanical and acidic challenges over time, whereas protocols without polymeric sealing films leave the desensitizing layer directly exposed from the outset. In this context, the present study aimed to compare the efficacy of three desensitizing protocols for dentin hypersensitivity (DH) pain control and their impact on oral health–related quality of life (OHRQoL) up to 180 days.

The null hypothesis of this study was that no statistically significant differences would be observed among the tested protocols in terms of dentin hypersensitivity pain reduction and oral health–related quality of life outcomes during the 180-day follow-up period.

## Materials and methods

### Ethics approval and protocol registration

The clinical trial project was reviewed and approved by the Ethics and Research Committee of the Federal University of Uberlândia (UFU) (CAAE 74756023.9.0000.5152) on 18 November 2023, in accordance with Resolution No. 466/2012 of the Brazilian Health Council. Participants were informed of all relevant aspects of the study and invited to participate. Informed consent was obtained through the signing of a Consent Form. Following approval, the project was registered in the Brazilian Clinical Trials Registry (RBR-109h3wcv) on 05 February 2024. The paper was written following the Consolidated Standards of Reporting Trials (CONSORT) guidelines.

### Trial design, settings, and location of data collection

This study was a randomized, controlled, split-mouth clinical trial conducted from June 2024 to April 2025 at the dental clinics of the School of Dentistry, UFU, Brazil.

### Sample size calculation

The sample size was determined a priori based on dentin hypersensitivity (DH) pain scores at 6 months reported by Forouzande et al. [23], who assessed pain using a 0–10 visual analogue scale (VAS). In that trial, the lowest and highest 6-month DH pain scores were 2.43 ± 1.77 and 4.25 ± 2.51, respectively, reported as mean ± standard deviation (SD) on the VAS. Using these parameters to estimate the expected between-protocol difference, the required sample size was calculated using a one-way ANOVA (independent samples), with α = 0.05, 80% power, and an anticipated dropout rate of 10%. The minimum required sample was 29 participants; therefore, 33 participants were enrolled to compensate for potential losses to follow-up, totaling 99 treated teeth.

### Recruitment

Participant recruitment was carried out through advertisements on social media platforms, including Instagram and WhatsApp. The final study sample consisted of patients receiving care at the undergraduate dental clinics of the School of Dentistry, Federal University of Uberlândia (FOUFU), as well as students from the institution and external individuals who expressed interest in participating in the research.

To be considered eligible, participants were required to present a clinical diagnosis of moderate to severe DH, defined as a pain score greater than 3 [24] in at least three teeth, according to the Numeric Rating Scale (NRS). Additional inclusion criteria included satisfactory oral hygiene and the absence of active carious lesions or periodontal disease.

Participant selection was based on a detailed clinical examination and a comprehensive anamnesis, both conducted during routine care in the undergraduate clinics. Volunteers were recruited on a first-come, first-served basis as they presented for screening. The initial evaluation session, held at the FOUFU clinical facilities, included a thorough assessment of the participants’ systemic and oral health conditions. Individuals who met all eligibility criteria received detailed information regarding the study objectives, procedures, risks, and potential benefits.

The informed consent form was presented and obtained by a responsible researcher prior to the participant’s inclusion in the study. Only those who provided formal written consent were enrolled. The initial screening was conducted by a second researcher. Individuals who declined to participate were referred for standard institutional dental care, without any compromise in the quality of treatment provided.

### Eligibility criteria

The inclusion criteria were as follows: Patients aged between 18 and 60 years with moderate to severe DH (pain score > 3), presenting with cervical tooth structure loss of less than 1 mm in at least 3 teeth.

The exclusion criteria were also defined: Presence of DH caused by caries or defective restorations; patients undergoing orthodontic treatment; patients undergoing tooth whitening; presence of spontaneous pain in the hypersensitive tooth, indicative of pulpitis; presence of periodontal disease; use of extensive prostheses; patients with severe occlusal disharmony; patients with uncontrolled gastroesophageal reflux disease; patients taking prescription medications that affect salivary flow or anti-inflammatory drugs; pregnant or breastfeeding women; smokers.

### Examiner calibration

All intervention protocols (GLU, GAS, and GAR) were applied by a single operator (J.M.M.). The materials used are listed in Table 1. Operator calibration consisted of standardized training and supervised practice prior to participant treatment. An experienced clinician (G.R.S.) first performed the complete application procedures for each protocol, demonstrating every step in detail. The operator then performed two applications per protocol under supervision. Technique deviations were identified, discussed, and corrected until the operator demonstrated consistent adherence to the study procedures. Only after this calibration process was completed was the treatment phase initiated.

**Table 1:** Materials used in the execution of DH

Material (generic name; trade name)	Manufacturer	Composition
3% potassium nitrate desensitizing gel; UltraEZ	Ultradent, South Jordan, UT, USA	Glycerol, polyacrylic acid, polyethylene glycol, potassium nitrate, sodium hydroxide.
Glutaraldehyde/HEMA desensitizer; Gluma Desensitizer	Kulzer, Hanau, Germany	HEMA monomer (hydroxyethyl methacrylate), desensitizing agent glutaraldehyde, and purified water.
Universal adhesive system; Ambar Universal APS	FGM, Joinville, SC, Brazil	MDP (10-methacryloyloxydecyl dihydrogen phosphate), methacrylate monomers, photoinitiator complex (APS), co-initiators, and stabilizers. Inactive ingredients: inert filler (silica particles) and vehicle (ethanol).
Bulk-fill flowable resin; Opus Bulk Fill Flow APS	FGM, Joinville, SC, Brazil	Urethane dimethacrylate monomers, stabilizers, camphorquinone, and co-initiator. Inactive ingredients: silanized inorganic silica fillers, stabilizers, and pigments.

### Randomization

To allocate participants to the experimental groups, a randomization list was generated at www.sealedenvelope.com using block randomization, with sets of three protocols per assessment point. The list was prepared by an independent researcher who was not involved in the intervention or outcome assessment phases. This researcher prepared 33 sets of opaque, sealed envelopes, each containing the randomized sequence of the three desensitizing protocols, identified as “GAS,” “GLU,” and “GAR”, to be applied to the designated teeth.

The application of the protocols in each participant followed a predefined pattern, respecting the standard anatomical order of the dental quadrants, beginning with the first quadrant and proceeding sequentially to the second, third, and fourth quadrants.

### Blinding

This study was designed as a randomized split-mouth clinical trial with participant blinding. Allocation was performed by an independent researcher who opened a sealed opaque envelope containing the randomized sequence immediately before the clinical procedure and informed the operator of the pre-established application order. Blinding of the operator was not feasible because the interventions required protocol-specific materials and application steps. Likewise, assessor blinding was not feasible because the applied materials could be identified during follow-up examinations. To maintain participant blinding, procedures were standardized and, when applicable, material application and light-curing steps were simulated to minimize participants’ perception of the allocated protocol. The allocation list was kept confidential, and the dataset provided for statistical analysis contained only coded group labels until the analyses were completed.

### Outcomes

The primary outcome was dentin hypersensitivity (DH) pain intensity measured with the Numeric Rating Scale (NRS; 0–10) in response to a standardized air-blast stimulus, assessed at baseline, immediately after the intervention, and at 7, 30, 90, and 180 days. Secondary outcomes were NRS pain intensity in response to a standardized tactile stimulus at the same time points; periodontal parameters (PD, GR, BOP, VP, PS, gingival phenotype, and KMH); and oral health–related quality of life assessed with OHIP-14 at baseline, 90, and 180 days.

### Initial assessment

#### Assessment of dentin hypersensitivity using standardized air-blast and tactile stimuli

After participant selection, baseline dentin hypersensitivity (DH) was assessed in each eligible tooth using a standardized air-blast (air-jet) stimulus and a tactile stimulus. To familiarize participants with the procedure and establish an individual reference for a non-hypersensitive response, the air-blast was first applied to a tooth without DH.

The air-blast stimulus was delivered with a three-way air–water syringe positioned approximately 1 cm from the cervical region of the test tooth. The air stream was directed toward the cervical surface and applied for 2 s. Adjacent teeth were isolated with cotton rolls to minimize false-positive responses.

Tactile sensitivity was evaluated using a UNC-15 periodontal probe (University of North Carolina-15). The probe tip was gently moved in a mesiodistal direction across the cervical surface using consistent, controlled pressure.

After each stimulus, participants rated pain intensity using the Numerical Rating Scale (NRS; 0–10), where 0 indicates no pain and 10 indicates the worst pain imaginable. Scores were recorded immediately after each stimulus and used for subsequent comparisons.

### Assessment of periodontal parameters

Using a UNC-15 manual periodontal probe, the following parameters were evaluated prior to the intervention: Probing depth (PD) – measured as the distance between the gingival margin and the apical extent of the gingival sulcus; Gingival recession (GR) – presence or absence; Bleeding on probing (BOP) – presence or absence at the study site; Visible plaque (VP) – presence or absence at the study site; Probing sensitivity (PS) – presence or absence. Additionally, the following parameters were recorded: gingival phenotype (thin or thick) and keratinized mucosa height (KMH).

### Assessment of oral Health–Related quality of life

The Oral Health Impact Profile questionnaire (OHIP-14) [25] was administered prior to the intervention protocols, with the aim of assessing how DH affected patients’ quality of life (Table 2). This baseline evaluation provided a reference point for measuring potential improvements in psychosocial and functional dimensions throughout the clinical follow-up.

**Table 2:** Domains and questions in the OHIP-14

OHIP-14 questionnaire	
Domain	Questions
Functional limitation	Have you had trouble pronouncing words? Have you felt that your sense of taste has worsened?
Physical pain	Have you had painful aching in your mouth? Have you found it uncomfortable to eat any foods?
Psychological discomfort	Have you been worried? Have you been nervous?
Physical disability	Has your diet been unsatisfactory? Have you had to interrupt meals?
Psychological disability	Have you found it difficult to relax? Have you been embarrassed?
Social disability	Have you been irritated with other people? Have you had difficulty doing your usual jobs?
Handicap	Have you felt that life in general was less satisfying? Have you been totally unable to function?

### Intervention

All treatment protocols were performed using products according to the manufacturers’ instructions, with the operator strictly following the guidelines provided in the product inserts. Patients underwent application of the different associative desensitizing protocols in a single clinical session. Initially, prophylaxis was performed on the hypersensitive teeth using pumice and water, followed by relative isolation with a lip retractor, cotton rolls, and continuous suction. A retraction cord (#000) (Ultradent; South Jordan, UT, USA) was then placed, and the desensitizing agents were applied according to the manufacturers’ recommendations (Table 3). After completion of the protocol, the retraction cord was removed along the tooth’s long axis.

**Table 3:** Desensitization protocols

Group	Protocol
GLU	A 3% potassium nitrate desensitizing gel (UltraEZ; Ultradent, South Jordan, UT, USA) was applied to the cervical third for 10 min, with gentle massage every 2 min, and then removed with a water spray. Next, a glutaraldehyde/HEMA desensitizer (Gluma Desensitizer; Kulzer, Hanau, Germany) was applied with a disposable applicator and rubbed for 30 s, followed by air-drying for 10 s and rinsing for 30 s.
GAS	A 3% potassium nitrate desensitizing gel (UltraEZ; Ultradent, South Jordan, UT, USA) was applied to the cervical third for 10 min, with gentle massage every 2 min, and then removed with a water spray. Next, a glutaraldehyde/HEMA desensitizer (Gluma Desensitizer; Kulzer, Hanau, Germany) was applied with a disposable applicator and rubbed for 30 s, followed by air-drying for 10 s and rinsing for 30 s. A universal adhesive system (Ambar Universal APS; FGM, Joinville, SC, Brazil) was actively applied to the cervical third for 10 s, followed by a second application for an additional 10 s; a moderate air stream was applied for 10 s, excess material was removed, and the adhesive was light-cured for 20 s using an LED curing unit (Valo Grand; Ultradent, South Jordan, UT, USA). Excess material was again removed with a scalpel blade no. 12.
GAR	A 3% potassium nitrate desensitizing gel (UltraEZ; Ultradent, South Jordan, UT, USA) was applied to the cervical third for 10 min, with gentle massage every 2 min, and then removed with a water spray. Next, a glutaraldehyde/HEMA desensitizer (Gluma Desensitizer; Kulzer, Hanau, Germany) was applied with a disposable applicator and rubbed for 30 s, followed by air-drying for 10 s and rinsing for 30 s. A universal adhesive system (Ambar Universal APS; FGM, Joinville, SC, Brazil) was actively applied to the cervical third for 10 s, followed by a second application for an additional 10 s; a moderate air stream was applied for 10 s, excess material was removed, and the adhesive was light-cured for 20 s using an LED curing unit (Valo Grand; Ultradent, South Jordan, UT, USA). Finally, a bulk-fill flowable resin (Opus Bulk Fill Flow APS; FGM, Joinville, SC, Brazil) was applied to the cervical third, excess material was removed, and light curing was performed for 40 s; excess material was again removed with a scalpel blade no. 12.

### Immediate Post-Intervention assessment and longitudinal Follow-Up

Immediately after the application of the desensitizing agent, the operator was replaced by the examiner, who assessed the level of DH in each tooth using the Numerical Rating Scale (NRS), based on air jet and tactile stimuli, as previously described in the “Initial Assessment” section.

DH was monitored longitudinally at 7, 30, 90, and 180 days through repeated assessments using the same stimuli (air jet and tactile), as well as the following periodontal parameters: PD, GR, BOP, VP, and PS. The Oral Health Impact Profile (OHIP-14) questionnaire was administered before the intervention and again at 90 and 180 days after treatment, in order to measure the impact of the desensitizing protocols on patients’ quality of life.

### Statistical analysis

The data generated from the dependent variables in this study were continuous numerical (PD, KMH), dichotomous (GR, BOP, VP, PS, gingival phenotype, and dental arch alignment), or ordinal categorical (NRS from air jet and probe stimuli, and OHIP-14). Numerical data were not normally distributed, as determined by the Shapiro-Wilk test. The primary outcomes (NRS to air-jet and tactile stimuli) were analyzed using generalized linear mixed models (GLMMs) with fixed effects for treatment, time, and the treatment × time interaction, and random effects to account for clustering within participants inherent to the split-mouth repeated-measures design. Given the ordinal nature of NRS scores, an ordinal (cumulative logit) mixed model was used for NRS. Therefore, the effects of the independent variables (time and treatment) were evaluated using generalized linear mixed models for parameters assessed at more than one time point (PD, GR, BOP, VP, PS, NRS from probe and air stimuli). The numerical parameter assessed at only one time point (KMH) was compared between groups using the Kruskal-Wallis test followed by Dunn’s post-hoc test. Dichotomous parameters evaluated at a single time point (arch alignment and gingival phenotype) were compared using Cochran’s Q test. OHIP-14 scores, which considered only the independent variable time, were analyzed using the Friedman test followed by Dunn’s post-hoc test. All statistical tests were conducted at a significance level of 5%. Inferential analyses were performed using SPSS version 27 (IBM, Armonk, NY, USA) and Jamovi software (The jamovi project, Sydney, Australia) by the statistician responsible for the analyses (G.J.P.L.O.).

## Results

**Fig. 1:**
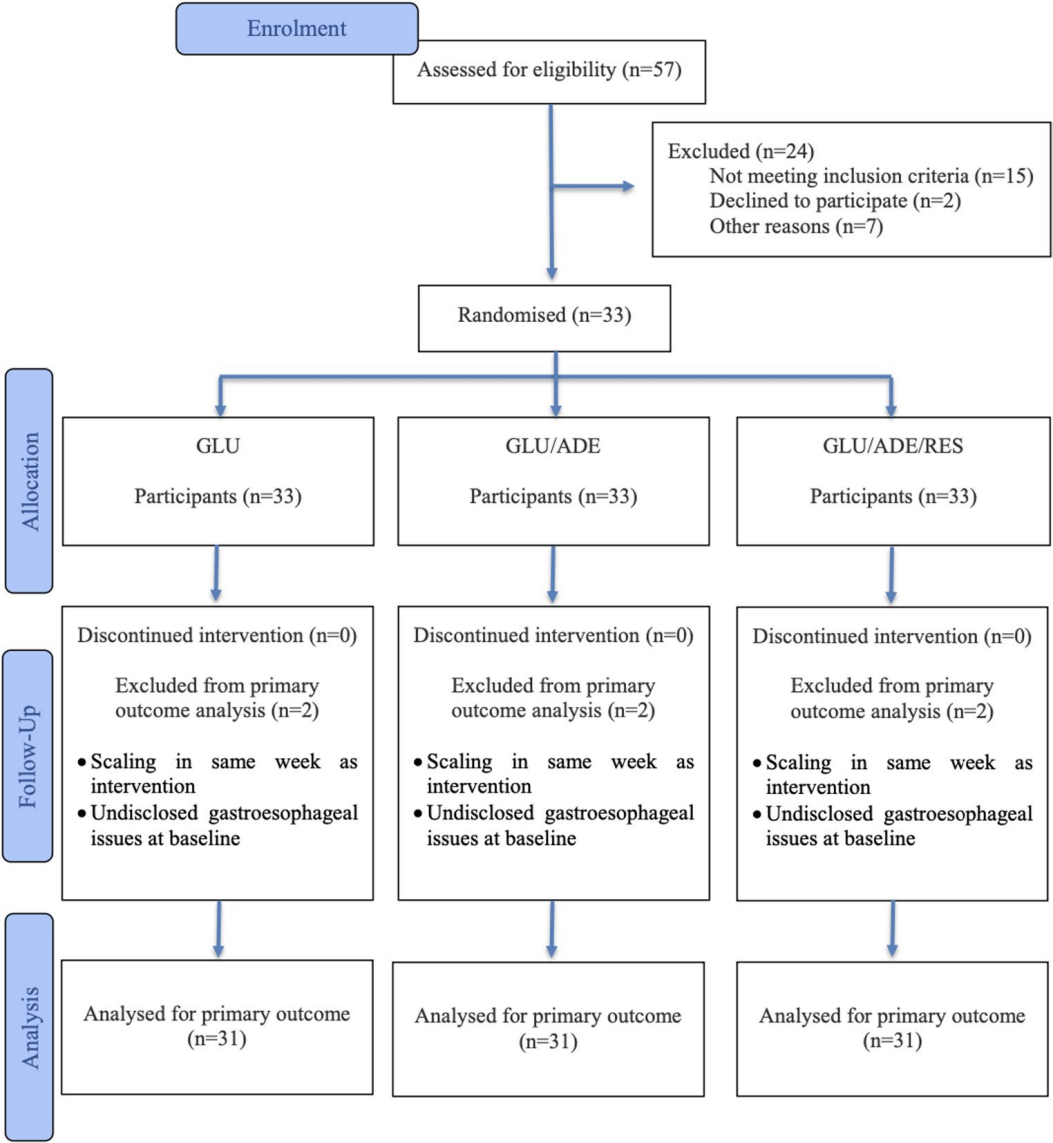
Flow diagram recommended by CONSORT 2025, identifying the number of individuals approached, enrolled, and followed up to 180 days, and the primary outcome analysis set (mITT). As this is a split-mouth study, exclusion of a single participant from the primary outcome analysis results in three desensitized teeth not included in the primary outcome analysis

A total of 57 individuals were assessed for eligibility; 24 were excluded (15 did not meet the inclusion criteria, 2 declined to participate, and 7 were unable to attend the scheduled visits). Thirty-three participants (99 teeth) were randomized and received the assigned interventions, with no losses to follow-up. For the primary outcome, two participants were excluded from the analysis due to post-randomization protocol/eligibility issues (supragingival scaling during the intervention week; previously unreported gastroesophageal disorder). Thus, the primary outcome was analyzed in 31 participants (93 teeth) (mITT) (Fig. 1).

Of the 31 participants, 90.3% were female and 9.7% were male; most were aged 18–29 years (80.6%) (Table 4). Among the 93 treated teeth, incisors accounted for 42.0%, followed by premolars (32.2%), canines (18.3%), and molars (7.5%); 63.4% were in the maxilla and 36.6% in the mandible (Table 4).

**Table 4:** Characteristics of the participants and teeth included in the study

Participant Characteristics	Number (%)
Sex	
Male	3 (9,7%)
Female	28 (90,3%)
Age (Years)	
18–29	25 (80,6%)
30–39	1 (3,2%)
39–49	1 (3,2%)
> 49	4 (13%)
Tooth distribution	
Incisors	39 (42%)
Canines	17 (18,3%)
Premolars	30 (32,2%)
Molars	7 (7,5%)
Arch location distribution	
Maxilla	59 (63,4%)
Mandible	34 (36,6%)

GLMMs showed a significant effect of time for both the air jet (*p* < 0.001) and tactile stimulus tests (*p* < 0.001). No statistically significant differences were found between treatment groups (air jet: *p* = 0.910; tactile stimulus: *p* = 0.681), and no time × treatment interaction was detected (air jet: *p* = 0.341; tactile stimulus: *p* = 0.738). All interventions (GLU, GAS, and GAR) reduced NRS pain scores in response to both stimuli, and this effect was maintained from the immediate post-intervention assessment through the 180-day follow-up period (Table 5).

None of the treatment protocols had an impact on the evaluated periodontal parameters (PD, KMH, GR, BOP, VP, PS, dental alignment, and gingival phenotype), regardless of the evaluation period (Table 6). Finally, DH control led to a statistically significant improvement in patients’ quality of life, as all domains of the OHIP-14 questionnaire showed improved scores at both T90 and T180 compared to baseline (Table 7).

**Table 5:** Median and interquartile range data for dentin hypersensitivity parameters evaluated across all groups and experimental time points

Parameter	Treatment	BAS*	IM	T7	T30	T90	T180	*p* value**
Air jet	GLU	5.00 (4.00–7.00)	1.00 (0.00–2.00)	1.00 (0.00–2.00)	1.00 (0.00–2.00)	0.00 (0.00–2.00)	1.00 (0.00-2.50)	< 0.001
	GAS	5.00 (4.50-7.00)	1.00 (0.00–3.00)	1.00 (0.00–3.00)	0.00 (0.00–3.00)	0.00 (0.00-1.50)	1.00 (0.00–3.00)	< 0.001
	GAR	5.00 (4.00-6.50)	2.00 (0.00–3.00)	1.00 (0.00–2.00)	1.00 (0.00–2.00)	1.00 (0.00–2.00)	1.00 (0.00–4.00)	< 0.001
*p* value***	0.910							
Tactile stimulus	GLU	0.00 (0.00-3.50)	0.00 (0.00–0.00)	0.00 (0.00–0.00)	0.00 (0.00–0.00)	0.00 (0.00–0.00)	0.00 (0.00–0.00)	< 0.001
	GAS	2.00 (0.00-4.50)	0.00 (0.00–1.00)	0.00 (0.00–0.00)	0.00 (0.00–0.00)	0.00 (0.00–0.00)	0.00 (0.00–0.00)	< 0.001
	GAR	2.00 (0.00–5.00)	0.00 (0.00–0.00)	0.00 (0.00–0.00)	0.00 (0.00–0.00)	0.00 (0.00–0.00)	0.00 (0.00–1.00)	< 0.001
*p* value***	0.681							

*GLU* 3% potassium nitrate + Gluma, *GAS* GLU + universal adhesive system, *GAR* GAS + bulk-fill flowable resin, *BAS* Baseline. *p < 0.05 – Time points with significantly higher NRS values compared to other follow-up periods. Generalized linear mixed model (GLMM). ** Row comparison *** Column comparison

**Table 6:** Median, interquartile Range, and frequency data of periodontal parameters evaluated across all groups and experimental time points

Parameter	Treatment	Presence	BAS	T7	T30	T90	T180
PD	GLU	-	1.00 (1.00-1.50)	1.00 (1.00–2.00)	1.00 (1.00–2.00)	1.00 (1.00–2.00)	1.00 (1.00–2.00)
	GAS	-	1.00 (1.00–2.00)	1.00 (1.00–2.00)	1.00 (1.00–2.00)	1.00 (1.00–2.00)	1.00 (1.00–2.00)
	GAR	-	1.00 (1.00–2.00)	1.00 (1.00–2.00)	1.00 (1.00–2.00)	1.00 (1.00–2.00)	1.00 (1.00–2.00)
KMH	GLU	-	3.00 (2.00–4.00)	-	-	-	-
	GAS	-	3.00 (2.00-3.50)	-	-	-	-
	GAR	-	3.00 (2.00–3.00)	-	-	-	-
GR	GLU	Yes	14	14	14	14	14
		No	17	17	17	17	17
	GAS	Yes	15	15	15	15	15
		No	16	16	16	16	16
	GAR	Yes	17	17	17	17	17
		No	14	14	14	14	14
BOP	GLU	Yes	1	1	3	1	0
		No	30	30	28	30	31
	GAS	Yes	1	2	2	1	0
		No	30	29	29	30	31
	GAR	Yes	1	0	1	0	0
		No	30	31	30	31	31
VP	GLU	Yes	1	0	1	1	2
		No	30	31	30	30	29
	GAS	Yes	1	1	2	1	3
		No	30	30	29	30	28
	GAR	Yes	1	0	1	1	2
		No	30	31	30	30	29
SP	GLU	Yes	1	1	3	1	0
		No	30	30	28	30	31
	GAS	Yes	1	2	2	1	0
		No	30	29	29	30	31
	GAR	Yes	1	0	1	0	0
		No	30	31	30	31	31
Gingival Phenotype	GLU	Thin	12	-	-	-	-
		Thick	19	-	-	-	-
	GAS	Thin	12	-	-	-	-
		Thick	19	-	-	-	-
	GAR	Thin	12	-	-	-	-
		Thick	19	-	-	-	-

*PS* Probing Depth, *KMH* Keratinized Mucosa Height, *GR* Gingival Recession, *BOP* Bleeding on Probing, *VP* Visible Plaque, *SP* Sensitivity to Probing, *GLU* 3% potassium nitrate + Gluma, *GAS* GLU + universal adhesive system, *GAR *GAS + bulk-fill flowable resin, *BAS* Baseline

**Table 7:** Median and interquartile range data for the 14 items of the OHIP-14 questionnaire, divided into 7 domains with 2 items each, assessed at baseline, 90 days, and 180 days. *p < 0.05 – Time points with significantly better quality of life compared to baseline (T0). Friedman test with dunn’s post hoc analysis

Baseline	Q1 + Q2	Q3 + Q4	Q5 + Q6	Q7 + Q8	Q9 + Q10	Q11 + Q12	Q13 + Q14	Q1 – Q14
	0.00 (0.00–1.00)	5.00 (4.00–6.00)	4.00 (2.00-5.50)	1.00 (0.00–3.00)	0.00 (0.00–1.00)	0.00 (0.00-1.50)	0.00 (0.00–1.00)	13.00 (6.50–18.00)
T90^*^	0.00 (0.00–0.00)	1.00 (0.00–2.00)	2.00 (1.00–4.00)	0.00 (0.00–0.00)	0.00 (0.00–0.00)	0.00 (0.00–0.00)	0.00 (0.00–0.00)	4.00 (2.00–7.00)
T180^*^	0.00 (0.00–0.00)	2.00 (1.50-3.00)	0.00 (0.00–2.00)	0.00 (0.00–0.00)	0.00 (0.00-0.50)	0.00 (0.00–0.00)	0.00 (0.00–0.00)	4.00 (1.50-6.00)
P value	0.001	< 0.001	< 0.001	< 0.001	0.047	0.035	0.016	< 0.001

*T* Time, *Q *Question

## Discussion

This randomized, split-mouth clinical trial evaluated a sequential, single-session approach combining neural modulation (3% potassium nitrate) with a glutaraldehyde/HEMA desensitizer and, in the experimental protocols, additional surface sealing steps (universal adhesive and flowable bulk-fill resin). The hypothesis was that the addition of these sealing steps could provide an incremental benefit in controlling dentin hypersensitivity symptoms under oral challengs. However, during the 180-day follow-up period, no evidence of differences among protocols in pain outcomes was found, and the null hypothesis was not rejected.

Potassium nitrate gel was selected as the neural agent, due to its wide clinical use and proven efficacy in reducing DH [26–31]. Gluma, in turn, was chosen as the chemical tubule-occluding agent for its effectiveness in providing immediate sensitivity relief, as shown in systematic reviews and randomized clinical trials. It has been reported to outperform fluoride varnishes and self-etching systems, with a sustained effect up to 30 days after a single application [32]. For instance, Parreiras et al. reported a significant reduction in DH following dental bleaching when potassium nitrate and Gluma were used in combination [33]. However, the persistence of desensitizing effects reported in the literature varies across agents and clinical settings. In the present trial, the baseline protocol (GLU) demonstrated sustained pain reduction within the 180-day follow-up, and the addition of adhesive and resin surface-sealing steps did not provide measurable additional benefit over this period [16, 31, 34].

Self-etch adhesive systems have been used as a surface-sealing approach aiming to improve the persistence of tubule occlusion [18, 35]. These systems are capable of forming an acid-base resistant zone, reducing dentin permeability [36–38]. Seventh-generation, all-in-one adhesives stand out for their simplified application and effective penetration into demineralized dentin [32]. Studies such as that by Askari and Yazdani (2019) demonstrated that dentin adhesives promote immediate and long-lasting symptom relief, outperforming alternative agents such as those based on propolis [39].

The second approach investigated consisted of the resin coating technique, which combines the adhesive system with the application of a thin layer of flowable resin composite [40]. As demonstrated by Nikaido et al. (2018), this technique enhances dentin sealing, reduces pulpal irritation, and reinforces the ABRZ, increasing resistance against acid-base challenges in the oral environment [41]. Despite the theoretical and potential benefits attributed to both surface-sealing strategies, the data from this study, after 180 days of follow-up, did not reveal statistically significant differences between groups (Table 5). Longer follow-up periods have been reported in the literature for dentin hypersensitivity interventions (including 12-month outcomes), and our findings should therefore be interpreted within the predefined 180-day follow-up of the present trial.

The stability of periodontal parameters throughout the follow-up period also deserves mention, especially considering the use of thicker restorative materials, such as flowable resin composite. None of the evaluated protocols led to significant changes in probing depth, gingival bleeding, biofilm presence, or gingival recession indices, supporting the clinical safety of the materials used (Table 6). Although in vitro studies have suggested potential cytotoxicity of Gluma [33, 42], the clinical data obtained in this study did not demonstrate relevant adverse effects. The application of adhesive materials near the gingival sulcus, when performed with technical rigor and in accordance with minimally invasive principles, was compatible with periodontal tissues integrity. Recent evidence indicates that gingival response is more closely related to marginal adaptation quality, proper polishing, and the absence of subgingival excess, rather than the isolated chemical composition of the materials [43, 44]. These observations indicate that periodontal parameters remained stable during follow-up when the materials were applied with careful technique and excess removal, while no additional clinical benefit in DH pain control was observed within the study period.

Another relevant finding was the statistically significant improvement in oral health-related quality of life (OHRQoL) across all groups, as measured by the OHIP-14 instrument at 90 and 180 days (Table 7). This questionnaire, validated and widely used in the literature [45, 46], showed that the tested protocols provided relevant clinical benefits in functional, psychological, and social domains. These findings reinforce the importance of systematically incorporating patient-reported outcomes in clinical trials on DH, as they capture subjective dimensions often not detected through conventional clinical assessments. The use of OHRQoL instruments strengthens study methodology and promotes a patient-centered dental practice, aligned with the principles of evidence-based medicine.

Despite its methodological robustness, this study has limitations. Pain perception varies between individuals and, although sensitivity testing was standardized and performed by a single examiner, variations in air pressure from the triple syringe could not be fully controlled. Moreover, the 180-day follow-up period, while clinically relevant, may be insufficient to characterize longer-term performance of surface-sealing materials under oral challenges. Longer follow-up may be informative to further characterize coating persistence and symptom trajectories beyond the timeframe assessed in this trial.

In summary, the addition of adhesive and bulk-fill flowable resin surface-sealing steps did not provide any measurable incremental benefit over the baseline protocol for dentin hypersensitivity pain control over 180 days; importantly, the absence of added benefit despite increased procedural complexity is clinically meaningful, as it supports a simpler and more efficient approach when the goal is symptom control for up to 180 days.

## Conclusion

This randomized split-mouth trial indicates that adding a universal adhesive and a bulk-fill flowable resin to a potassium nitrate + glutaraldehyde/HEMA, based protocol does not improve patient-centered outcomes for dentin hypersensitivity pain control or oral health–related quality of life up to 180 days. These results support evidence-based selection of streamlined protocols when the clinical goal is symptom control and quality-of-life improvement within the evaluated period.

## Data Availability

All data generated or analyzed during this study are included in this article. Further enquiries can be directed to the corresponding author.
